# Activation of P2X7 receptor and NLRP3 inflammasome assembly in hippocampal glial cells mediates chronic stress-induced depressive-like behaviors

**DOI:** 10.1186/s12974-017-0865-y

**Published:** 2017-05-10

**Authors:** Na Yue, Huijie Huang, Xiaocang Zhu, Qiuqin Han, Yalin Wang, Bing Li, Qiong Liu, Gencheng Wu, Yuqiu Zhang, Jin Yu

**Affiliations:** 1 Department of Integrative Medicine and Neurobiology, School of Basic Medical Sciences, Institues of Brain Science, State Key Laboratory of Medical Neurobiology and Collaborative Innovation Center for Brain Science, Fudan University, Shanghai, 200032 China; 2 Department of Anatomy, Histology and Embryology, School of Basic Medical Sciences, Shanghai Medical College, Fudan University, Shanghai, 200032 China; 3 Key Laboratory of Medical Imaging Computing and Computer Assisted Intervention of Shanghai, Shanghai, 200032 China

**Keywords:** Depression, P2X7 receptor, eATP, Glia, NLRP3 inflammasome

## Abstract

**Background:**

In recent years, proinflammatory cytokine interleukin-1β (IL-1β) was considered to play a critical role in the pathogenesis of depression. In addition, P2X7 receptor (P2X7R), a member of the purinergic receptor family, which is predominantly present on microglia, as well as on astrocytes and neurons in lesser amounts in the central nervous system, was suggested to be involved in the processing and releasing of IL-1β. Here, we investigated the role of P2X7R in the pathogenesis of depression.

**Methods:**

Male Sprague-Dawley rats were subjected to chronic unpredictable stressors (CUS) for 3 weeks. At the end of week 1, 2, and 3, extracellular ATP, caspase 1, IL-1β, and components and activation of NLRP3 inflammasome (nucleotide-binding, leucine-rich repeat, pyrin domain containing 3) were evaluated as biomarker of neuroinflammation. In separate experiments, the rats were microinjected with P2X7R agonists ATP, BzATP, and saline into the hippocampus, respectively, or exposed to CUS combined with hippocampal microinjection with P2X7R antagonist, BBG and A438079, and saline, respectively, for 3 weeks, followed by exposed to forced swimming test and open-field test. Moreover, we also evaluated the depressive and anxiety-like behavior of *P2X7-*null mice in forced swimming test, open-field test, and elevated plus maze.

**Results:**

Along with stress accumulation, extracellular ATP, cleaved-caspase 1, IL-1β, and ASC were significantly enhanced in the hippocampus, but P2X7R and NLRP3 were not. Immunoprecipitation assay indicated that along with the accumulation of stress, assembly of NLRP3 inflammasome and cleaved caspase 1 in NLRP3 inflammasome were significantly increased. Moreover, antagonists of P2X7R, either BBG or A438079, prevented the development of depressive-like behaviors induced by chronic unpredictable stress in rats. Meanwhile, we could not observe any depressive-like or anxiety-like behaviors of *P2X7-*null mice after they had been exposed to CUS. The results implied that *P2X7* knockout could impede the development of depressive-like and anxiety-like behaviors induced by CUS. In contrast, chronic administration of agonists of P2X7R, either ATP or BzATP, could induce depressive-like behaviors.

**Conclusions:**

The activation of P2X7R and subsequent NLRP3 inflammasome in hippocampal microglial cells could mediate depressive-like behaviors, which suggests a new therapeutic target for the prevention and treatment of depression.

**Electronic supplementary material:**

The online version of this article (doi:10.1186/s12974-017-0865-y) contains supplementary material, which is available to authorized users.

## Background

Depression is a common mental disease associated with long-term morbidity, a high recurrence rate, and significant mortality [1]. It is now clear that chronic uncontrollable stress is the main cause for this disorder [2–4]; underlying mechanisms of stress or depression have not been fully elucidated. In the past decade, neuroinflammation, described by overproduction of inflammatory cytokines in the brain, has been recognized as an important mechanism of depression [5–7]. Depressed patients exhibited increased inflammatory cytokines, such as IL-1β, IL-6, and IFN-γ, in peripheral circulation and some brain regions [8–10]. Animal studies also demonstrate that exposure to stress increases IL-1β in several brain areas, including the hippocampus, a key area of the brain responsible for memory and emotion. [11–13]. Central administration of IL-1β produces several stress-like effects and pathological changes [12, 14–16]. Hippocampal tissue inflammation and, particularly, enhanced interleukin-1β (IL-1β) signaling may contribute to depression [17]. In contrast, blockade of the hippocampal IL-1β receptor, IL-1RI, abolishes the anhedonic behavioral effects of chronic unpredictable stress (CUS) [18, 19]. However, the mechanisms underlying the ability of stress to increase IL-1β and inflammatory responses in the hippocampus have not been determined.

Meanwhile, the role of ATP-gated transmembrane cation channel P2X7 receptor in the neuroinflammation has received particular attention due to its widespread involvement as a key regulatory element of IL-1β maturation [20–22]. It has been reported that in the brain, P2X7R is an ionotropic receptor located predominantly on microglia and is activated in response to cellular danger signals, such as ATP [23]. In contrast, P2X7R antagonists inhibited IL-1β release from glial cells [24]. Furthermore, the *P2X7R* gene which is located on chromosome 12q24.31 has been identified as a susceptibility locus for affective disorders [25]. And recent studies have exhibited that P2X7R knockout may have an antidepressant-like effect in mice [26–28]. The emerging evidence indicates that P2X7R maybe plays a pivotal role in depression and mediates the IL-1β maturation, but the precise mechanisms associated with stress-induced hippocampal neuroinflammation merited further investigation.

Studies of peripheral immune cells demonstrated that activation of P2X7R induced oligomerization of NLRP3 with an adaptor protein (ASC [apoptosis-associated speck-like protein containing a CARD]) and pro-caspase-1 [29, 30]. The multiprotein complex named the inflammasome cleaves pro-caspase-1 to mature caspase-1, which subsequently causes the maturation of pro-IL-1β [31, 32]. In addition, recently, researches exhibited that NLRP3 inflammasome is activated in depressed animal models [33] and in depressive patients as well [34], which suggests that NLRP3 inflammasome might be a new target and offer new perspectives in the study of depression [34].

Moving from this evidence, the aim of the present investigation was to identify the pathways by which chronic stress increases IL-1β and the resulting depressive-like behaviors. We exhibited that with the accumulation of stressors, the concentration of eATP increased significantly. Meanwhile, the accumulation of stressors caused an increase in the assembly of NLRP3 inflammasome, a prominent downstream signal of P2X7R. Besides, the protein levels of ASC and cleaved-caspase 1 (p10), a component and product of NLRP3 inflammasome respectively, were increased after exposure to CUS. Also, the level of hippocampal IL-1β (p17), product of active caspase 1(p10), were upregulated with the accumulation of stressors.

## Methods

### Animals

Male Sprague-Dawley rats (180–200 g) and wild-type C57BL/6 and *P2X7*-null mice (male and female, 25–30 g) were housed in groups of 2–4 per cage under a 12-h light/dark cycle with ad libitum to access to food and water (except when indicated). Eight to 12 rats or mice per group were randomly chosen. The maintenance of rat and mouse colonies and all animal treatments and procedures were in accordance with NIH laboratory care standards and approved by the Experimental Animal Ethics Committee of Shanghai Medical College, Fudan University.

### CUS procedure

CUS is a rodent model of depression which animals are exposed to random sequence of mild stressors as previously described [35]. Rats were subjected to 6 different stressors: water deprivation (40 h), food deprivation (40 h), light-dark cycle reversal, hot environment (40 °C, 5 min), swimming in cold water (4 °C, 5 min), and cage shake (30 min). These stressors were performed every per day in a random order for 1, 2, and 3 weeks in different experiments (see Table 1). Mice were subjected to nine different stressors: food and water deprivation (24 h), light inversion (12 h), stroboscopic (12 h), hot environment (40 °C, 5 min), swimming in cold water (4 °C, 5 min), cage shake (30 min), wet bedding (6 h), cage tilt (2 h), and restraint (2 h). These stressors were performed two per day in a random order for 5 weeks (see Table 2).

**Table 1:** CUS procedure for rats

Week	Monday	Tuesday	Wednesday	Thursday	Friday	Saturday	Sunday
1	Hot environment 40 °C, 5 min	Water deprivation 24 h	Swimming in cold water 5 min	Cage shake 30 min	Light-dark cycle reversal	Hot environment 40 °C, 5 min	Food deprivation
2	Swimming in cold water 5 min	Light-dark cycle reversal	Hot environment 40 °C, 5 min	Water deprivation 24 h	Cage shake 30 min	Cage tilt 2 h Food deprivation 24 h	Swimming in cold water 5 min
3	Cage shake 30 min	Hot environment 40 °C, 5 min	Food deprivation 24 h	Light-dark cycle reversal	Water deprivation 24 h	Swimming in cold water 5 min	Hot environment 40 °C, 5 min

**Table 2:** CUS procedure for mice

Week	Monday	Tuesday	Wednesday	Thursday	Friday	Saturday	Sunday
1	Restraint 2 h Hot environment (40 °C, 5 min)	Cage tilt 2 h Food and water deprivation 24 h	Swimming in cold water 5 min	Cage shake 30 min Light inversion 12 h	Wet bedding 6 h Stroboscopic light 12 h	Cage tilt 2 h Hot environment (40 °C, 5 min)	Cage shake 30 min Food and water deprivation 24 h
2	Cage tilt 2 h	Swimming in cold water 5 min Light inversion 12 h	Restraint 2 h Stroboscopic light 12 h	Hot environment (40 °C, 5 min) Wet bedding 6 h	Restraint 2 h Hot environment (40 °C, 5 min)	Cage tilt 2 h Food and water deprivation 24 h	Swimming in cold water 5 min
3	Cage shake 30 min Light inversion 12 h	Wet bedding 6 h Stroboscopic light 12 h	Swimming in cold water 5 min hot environment (40 °C, 5 min)	Cage shake 30 min Food and water deprivation 24 h	Cage tilt 2 h	Swimming in cold water 5 min Light inversion 12 h	Restraint 2 h Stroboscopic light 12 h
4	Restraint 2 h Wet bedding 6 h	Restraint 2 h Hot environment (40 °C, 5 min)	Cage tilt 2 h Food and water deprivation 24 h	Swimming in cold water 5 min	Cage shake 30 min Light inversion 12 h	Wet bedding 6 h Stroboscopic light 12 h	Cage tilt 2 h Hot environment (40 °C, 5 min)
5	Cage shake 30 min Food and water deprivation 24 h	Swimming in cold water 5 min	Wet bedding 6 h Light inversion 12 h	Restaint 2 h Stroboscopic light 12 h	Hot environment (40 °C, 5 min) Wet bedding 6 h	Restraint 2 h Cage shake 30 min	Cage tilt 2 h Hot environment (40 °C, 5 min)

### Behavioral testing

Open-field test were performed as described before [36], rats or mice were placed into the center of a Plexiglas box (rats: 100 cm × 100 cm × 40 cm, mice: 50 cm × 50 cm × 40 cm, respectively) in a brightly lit room. During a 5-min session, animals were scored for the number of rearing and the distance traveled in the box. Animal behavior was recorded and subsequently analyzed using a video-tracking system (Shanghai Mobile Datum Information Technology Company, Shanghai, China).

For the forced swimming test, rats or mice were individually put into 18- or 15-cm-diameter glass cylinder filled to 30 or 20 cm with 23 ± 1 °C water, respectively, as we described before [37]. During analysis of the recordings, immobility was defined as the absence of all movement except motions required maintaining the animal’s head above the water. Struggling was defined as vigorous movements with forepaws breaking the water. Results were expressed as time (in seconds) that animals spent immobile or struggling during a 5-min session.

The elevated plus maze was shaped like a plus sign and consisted of a central platform (5 × 5 cm), two opposite open arms (30 cm × 5 cm), and two equal-sized opposite closed arms, elevated 50 cm from floor and illuminated by a dim light. Individual trials lasted for 5 min each and were recorded with a video-tracking system (Shanghai Mobile Datum Information Technology Company, Shanghai, China). Percentage of open-arm entries ([open entries]/[total entries] × 100) and open-arm time ([time in open arms]/[time in total arms] × 100) were calculated as we described in our previous paper [38].

All the behavioral tests were performed by an individual who was blinded to the animal’s treatment status.

### Stereotaxic microinjection

The rats were anesthetized with pentobarbital sodium and placed in a stereotaxic frame. A brain infusion cannula was bilaterally implanted in the hippocampus (AP = −3.8, ML = ±3 mm, DV = 3.5 mm, 1 μl, 0.5 μl min^−1^). Seven days after implantation, vehicle, ATP (100 nM), BzATP (10.5 nM), BBG (Brilliant Blue G, one of the safest dyes currently used, is a well-known P2X7 receptor antagonist,1 pM), or A438079 (a selective competitive antagonist for the human and rodent P2X7 receptor with good bioavailability and CNS penetration widely used in animal models of disease, 1.75 nM) was delivered into the hippocampus of free-moving rats for 21 days [39–49]. The behavioral test was conducted before the infusion on the first day and 30 min following the infusion on the 21th day. All compounds were dissolved in ACSF.

### Experimental procedures

Before starting every behavioral or pharmacological experiment, the mice or rats were randomly assigned to receive different treatments.

#### Experiment 1: change of eATP, P2X7R, NLRP3 inflammasome, astrocyte, and microglia in the hippocampus of rats submitted to CUS

Rats were exposed to one stressor every day for 1, 2, or 3 weeks. The stressors were used in a random order as mentioned above. Twenty-four hours after the last stressor, five rats of every group were randomly selected to be stereotaxically implanted with a microdialysis intracerebral guide cannula into the hippocampal DG zone. The hippocampal microdialysates were later collected through the cannula from the rats. The operational details were described below. Other rats of every group were immediately sacrificed to collect the brain tissue. Control animals were exposed to behavioral tests and not subjected to any stressors.

#### Experiment 2: effect of antagonists of P2X7R in model animals

Following exposure to the stressor of CUS 30 min later, the rats were microinjected with BBG (1 pM, Sigma/Aldrich, *n* = 11), A438079 (1.75 nM, Sigma/Aldrich, *n* = 13), and vehicle (*n* = 11), respectively, in the hippocampus every day for 3 weeks. Twenty-four hours after the last stressor, the rats were assessed for depressive-like behaviors in open-field test (OFT) and forced swimming test (FST).

#### Experiment 3: effect of agonists of P2X7R in normal animals

Rats received ATP (100 nM, Sigma/Aldrich, *n* = 12), BzATP (10.5 nM, Sigma/Aldrich, *n* = 12), vehicle (*n* = 12), or CUS treatment, respectively, for 3 weeks. All of the drugs were microinjected into the hippocampus once a day. The rats in the CUS group were exposed to one stressor in a random order every day. Twenty-four hours after the last stressor, the rats were assessed for depressive-like behaviors in OFT and FST.

#### Experiment 4: effect of CUS in *P2X7*-null mice

Wild-type C57BL/6 and *P2X7*-null mice (male and female) were exposed to a variety of stressors for 5 weeks. The stressors were used in a random order, as mentioned above. Twenty-four hours after the end of the CUS protocol, the mice were assessed for depressive-like behaviors in OFT, FST, and elevated plus maze test (EPM). Control animals were exposed to behavioral tests and not subjected to any stressors.

### Measurement of eATP concentration in the hippocampus

The microdialysis-intracerebral-guide cannula (Bioanalytical Systems, Inc., West Lafayette, USA) was implanted into the hippocampal DG zone (V 3.5 mm, AP 3.8 mm, ML 3.0). A microdialysis probe with a 2-mm membrane (Bioanalytical Systems, Inc., WestLafayette, USA) was then placed through the guide cannula into the DG region. Artificial cerebrospinal fluid (ACSF) with the ectonucleotidase inhibitor 6-*N*,*N*-diethyl-β-γ-dibromomethylene-D-adenosine-5-triphosphate FPL 67156 (ARL 67156 trisodium salt) was applied as the perfusate. The optimal perfusion flow-rate was set at 1 μl/min, and the sampling time for each rat was 2.5 h, 30 min after exposuring to the stressor of CUS. The ATP levels were determined using a bioluminescent ATP assay kit (Promega, Madison, WI, USA) and a luminometer (PerkinElmer) according to the manufacturer’s instructions. A calibration curve was obtained from standard ATP samples, and the luminescence of normal medium was considered to be the background ATP level.

### Immunohistochemical analysis

The brains were separated and post fixed in 4% PFA at 4 °C overnight and immersed in 20% sucrose (4% PFA as solvent) followed by 30% sucrose (in 0.1 M PBS). The brain samples were cut into 30-μm-thick sections (CM1850, Leica Microsystems, Wetzlar, Germany). Sections were incubated in 0.01 mol/L citrate buffer, pH 6.0 for high-temperature antigen retrieval. Tissues were blocked in 2% (wt/vol) BSA (Sigma) and then exposed overnight to the following primary antibody mixtures: anti-P2X7R (Abcam, 1:400), anti-GFAP (Thermo, 1:1000), or anti-Iba-1 (Wako, 1:1000) at 4 °C. Detection of primary antibodies was performed with secondary antibodies (donkey-anti-mouse, Alexa 594 conjugated,1:1000, Invitrogen, USA; donkey anti rabbit, Alexa 594 conjugated, 1:1000, Invitrogen, USA; Hoechst, 1: 1000, Beyotime, China) for 1 h in the dark. The sections were then washed five times with PBS in the dark. Immunofluorescent sections were observed with a Leica SP5 fluorescence microscope, using excitation wavelengths of 633 nm (helium/neon2, blue Cy5-labelling), 543 nm (helium/neon1, red Cy3-immunofluorescence), and 488 nm (argon, yellow-green Cy2-immunofluorescence), and images were captured with a CCD spot camera for data analysis. Three regions per hippocampus section and three sections per animal were counted by experimenters who were blind to the experiment design.

### Western blot analysis

The hippocampi of rats were homogenized in RIPA buffer (Thermo Scientific) with protease inhibitors (Beyotime). Protein samples were run on 12% tris-glycine SDS-PAGE gels, transferred to PVDF membrane (0.2 or 0.45 μm), and blotted with antibodies against P2X7R (1:200, Santa Cruz); Cryopyrin (NLRP3, 1:200, Santa Cruz); Caspase-1 (1:200, Santa Cruz); ASC (1:200, Santa Cruz); IL-1β (1: 1000, R&D System); or GAPDH (1:10000, KangCheng). Primary antibody incubation was performed overnight at 4 °C. Secondary antibodies (1:10000, Earthox) were incubated for 1 h at room temperature. The signal was captured on an ImageQuant LAS4000 mini image analyzer (GE Healthcare, Buckinghamshire, UK), and the band levels were quantified using Image J software (NIH, Bethesda, MD, USA).

### Quantitative real-time RT-PCR

Total RNA was isolated from hippocampi of rats using TRIzol reagent (Invitrogen) according to the manufacturer’s instructions. cDNA was synthesized by a PrimeScript Kit (Bio-Rad). Quantitative real-time PCR was performed by using gene-specific primers and SYBR Premix Ex Taq (Bio-Rad, California, USA). Oligonucleotide primers specific for rat are GFAP (F 5′GAGATGATGGAGCTCAATGACC 3′; R 5′TGGATCTCCTCCTCCAGCGA 3′); Iba-1 (F 5′ GGGATCAACAAGCACTTC 3′; R 5′ TATCTCCATTGCCATTCA3′); GAPDH (F 5′ CCCTTCATTGACCTCAACTAC 3′; R 5′ CTTCTCCATGGTGGTGAAGAC 3′). Relative mRNA expression levels were analyzed using the formula 2^−ΔΔCt^ method and normalized to the GAPDH ribosomal RNA.

### Immunoprecipitation (IP)

This test was performed as we described in our previous paper [50]. Hippocampus lysates (500 μg) were immunoprecipitated with 1 μg of anti-ASC antibody (Santa Cruz) overnight at 4 °C, then incubated with 30 μl of protein A agarose beads (Cell Signaling) at 4 °C for 4 h, and centrifuged at 12,000×*g* for 60 s. Protein complexes were washed five times with RIPA buffer, resuspended in 2× loading buffer, and heated at 95 °C for 10 min before western blot analysis by using the following antibodies: rabbit anti-ASC (1:1000, Santa Cruz), rabbit anti-NALP1 (1:200: Abcam), rabbit anti-NLRP3 (1:200, Santa Cruz), and rabbit anti-caspase-1 (1:200: Santa Cruz). Whole tissue lysate prepared for IP (50 μg) was used as an input, and homophytic IgG as the negative control. 

### Statistical analysis

All data were analyzed using SPSS 16.0 (SPSS Inc., Chicago, USA). The data collection and analysis were performed independently by two experimenters. Results are expressed as the mean ± standard error. Data were analyzed using one- or two-way analysis of variance (ANOVA) according to the factors introduced in the experimental design. Where F ratios were significant, post hoc comparisons were made using the Tukey post hoc test. Significance levels were set at *p* < 0.05.

## Results

### CUS causes extracellular ATP increment in the hippocampus in early stage

**Fig. 1:**
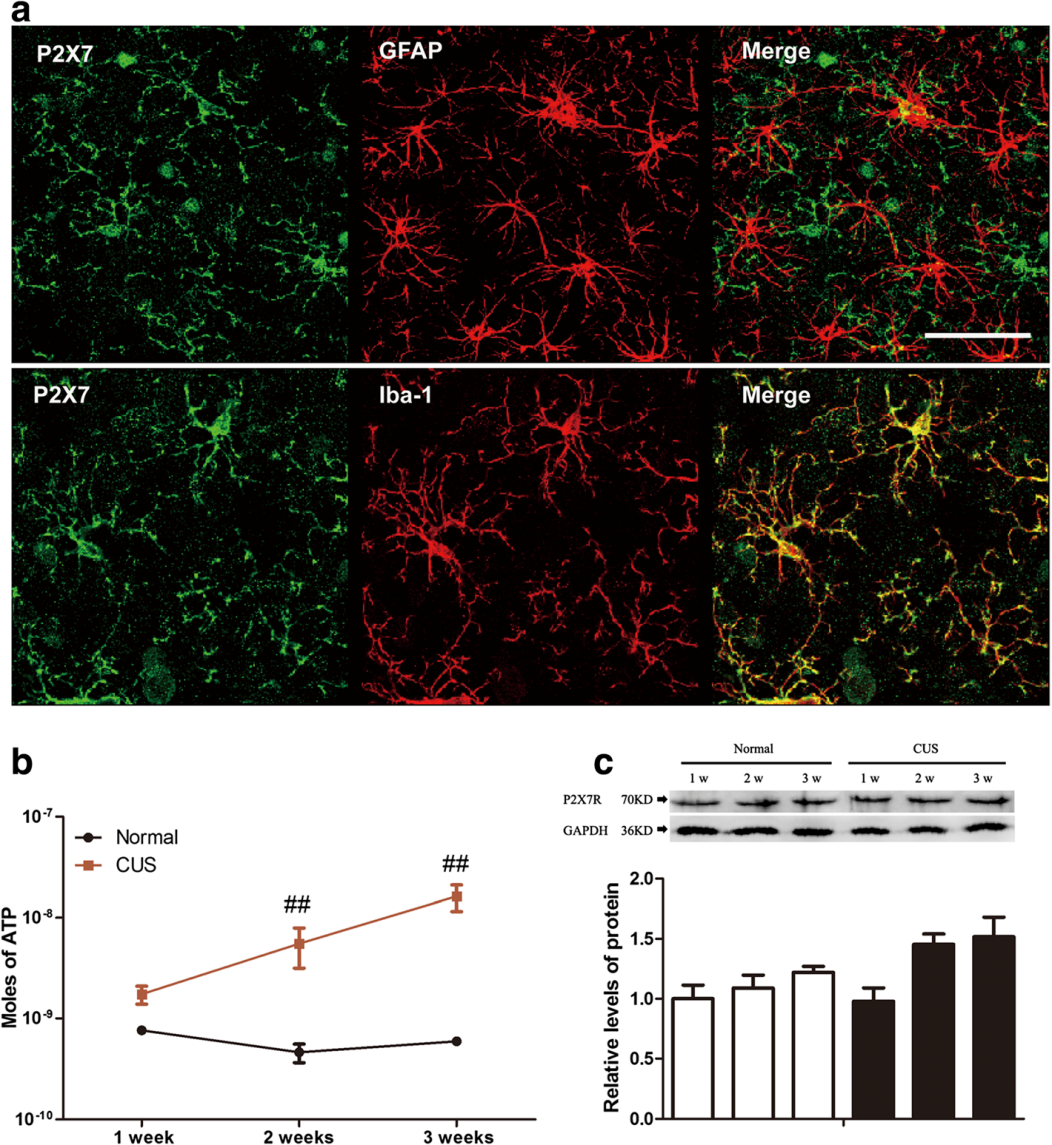
CUS increases ATP in microdialysates from the hippocampus but not levels of P2X7 receptor protein in the hippocampus. **a** Immunofluorescence staining of hippocampal sections from normal rats. P2X7R (*green*), Iba-1 (*red*), GFAP (*red*), DAPI (*blue*); scale bar, 50 μm. **b** Microdialysis samples were collected from the hippocampus of rats exposed to 1, 2, and 3 weeks of CUS, respectively, and analyzed for levels of ATP (*n* = 4 per group). **c** Western blots of P2X7R in the hippocampus from rats exposed to 1, 2, and 3 weeks of CUS, respectively. GAPDH was used as a loading control and the graphs represent the mean ± SEM (*n* = 4 per group). ^##^ *p* < 0.01 compared to normal rats at the same time point

We first detected the expression of P2X7R in normal rats’ hippocampus. The results showed that P2X7R were predominantly expressed by microglia, as well as astrocyte in less amounts (Fig.1a, Additional file 1: Figure S1 A–I). The next question is whether CUS causes eATP, the endogenous ligand of P2X7R to increase or induce the change of P2X7R in the hippocampus of rats. Thereby, we further detected the concentration of eATP and the expression of P2X7R in the rats’ hippocampus after they were exposed to 1, 2, and 3 weeks of CUS, respectively. As shown in Fig.1b, compared to the normal rats, the stressed rats showed a noteworthy increase in concentration of eATP in the hippocampus from the first weekend to the third weekend after being exposed to the CUS procedure. Meanwhile, we could not detect any difference in protein levels of P2X7R in the hippocampus between different groups (Fig. 1c).

### CUS causes the cleavage of caspase-1

**Fig. 2:**
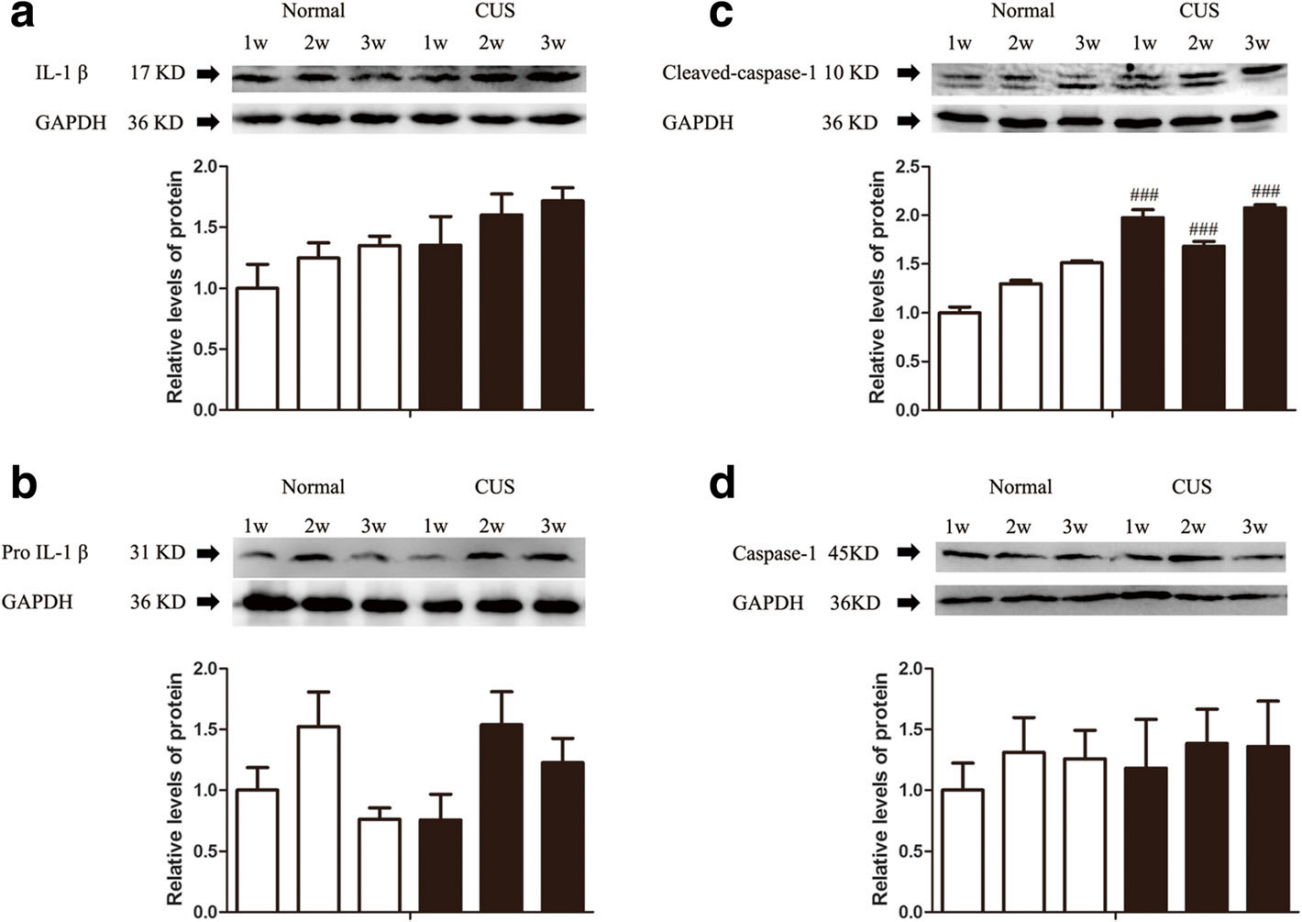
CUS increases levels of cleaved caspase-1 in the hippocampus of rats. Rats were exposed to CUS for 1, 2, and 3 weeks, respectively, and hippocampi were collected and analyzed for levels of (**a**) IL-1β (*n* = 4 per group), (**b**) pro-IL-1β (*n* = 4 per group), (**c**) cleaved caspase-1 (*n* = 4 per group), and (**d**) caspase-1 (*n* = 4 per group) using western blotting. Results are expressed as the mean ± SEM. ^###^ *p* < 0.001 compared to normal rats at the same time point

As we know, P2X7R activation by ATP stimulates caspase-1 activity which, in turn, leads to the regulated release of the cytokine interleukin-1 (IL-1β) from phagocytes [51]. We further investigated whether CUS can also cause cleavage of caspase-1 and consequent matured IL-1β increase along with the eATP augment. We found that matured IL-1β was slightly enhanced in the hippocampus of rats after exposed to 1, 2, and 3 weeks of CUS, respectively (Fig. 2a, b). Additionally, 1, 2, and 3 weeks of CUS significantly increased the protein level of caspase-1 p10 (cleaved-caspase-1) but not caspase-1 p45 (Fig. 2c, d) in the hippocampus of rats. These results suggest that stress induced capspase-1 activation and interleukin-1 maturation in the hippocampus of rats.

### CUS also induces NLRP3 inflammasome assembly in the hippocampus

**Fig. 3:**
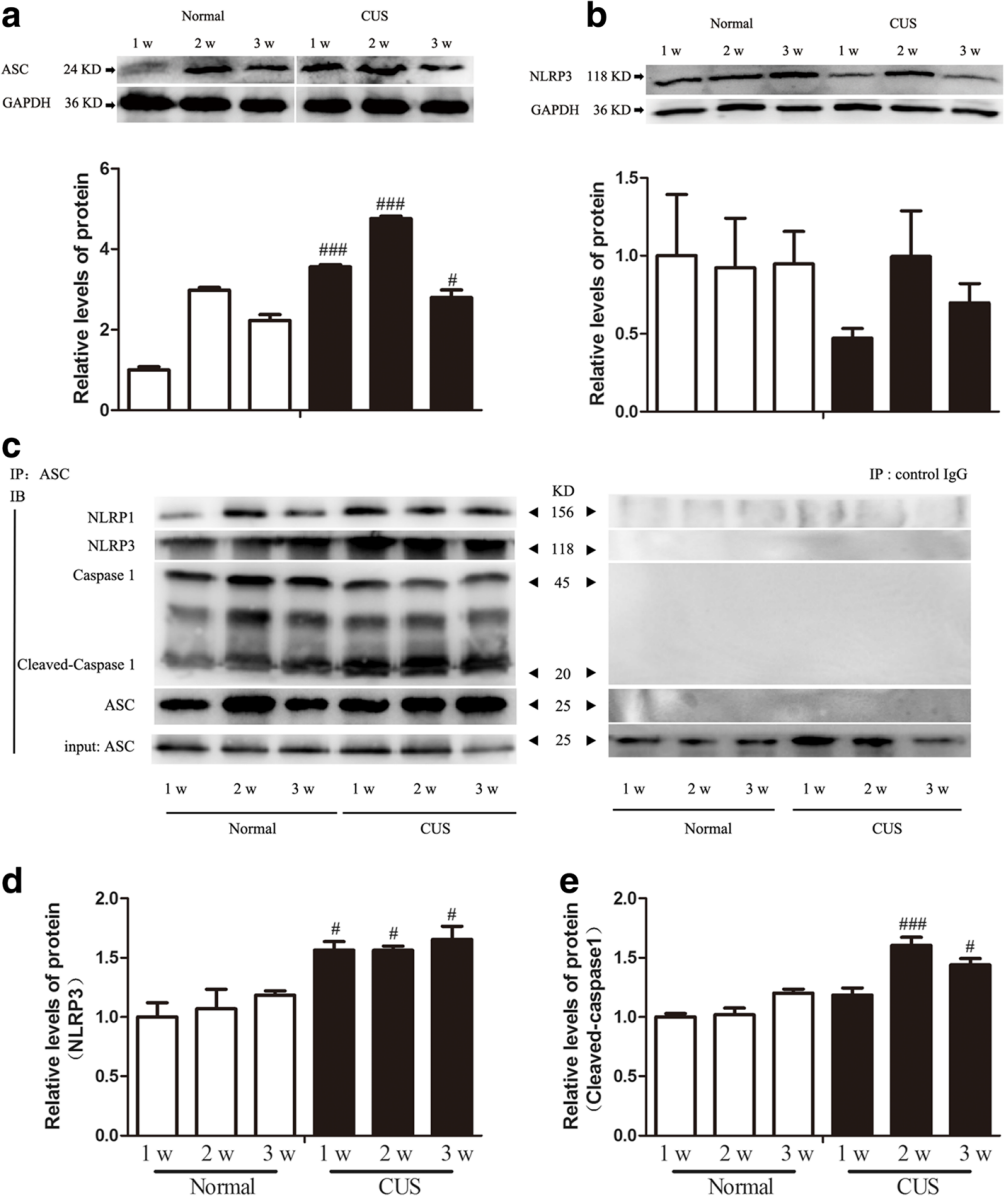
CUS increases levels of ASC and activates the NLRP3 inflammasome. **a**, **b** Rats were exposed to CUS for 1, 2, and 3 weeks, respectively, and hippocampi were collected and analyzed for levels of NLRP3 and ASC using western blotting. Quantification of the changes of NLRP3 and ASC between the groups (*n* = 4 per group). **c** Co-immunoprecipitation assay was operated to determine the assembly of NLRP1/3 inflammasome in the hippocampus of rats subjected to different treatments anti-ASC antibody coupled beads or IgG control using western blotting. **d**, **e** Quantification of the protein changes of NLRP3 and cleaved caspase-3 according to the co-immunoprecipitation assay results (*n* = 4 per group). ^#^ *p* < 0.05, ^###^ *p* < 0.001 compared to normal rats at the same time point

Many research studies have indicated that the assembly of inflammasome is the key pathway to activating caspase-1 [30, 32]. The inflammasome is a multiprotein complex, which was formed by NOD-like receptors’ (NLRs) family members, adaptor ASC (apoptosis-associated speck-like protein containing a CARD) and pro-caspase-1. Among NLRs, NLRP3 inflammasome were found to be activated or highly expressed in glial cells following brain injury or in several neurodegenerative diseases [52–55]. Besides, one of the most potent activators of the NLRP3 inflammasome is extracellular ATP acting at the P2X7 receptor (P2X7R) [29]. Therefore, we further detected the NLRP3 and ASC protein levels and the assembly of NLRP3 inflammasome in the hippocampus. We found that CUS could significantly increase the expression of ASC, but not NLRP3 protein level in the hippocampus (Fig. 3a, b). Moreover, we detected NLRP3, caspase-1, and the active p10 caspase-1 in the immunoprecipitated products of an ASC antibody (Fig.3c). And the subsequent western blot results showed that along with stress accumulation, the NLRP3 and cleaved caspase-1 in the IP product of an ASC antibody were significantly increased (Fig. 3c–e). The results demonstrate that the CUS promoted the assembly of NLRP3, ASC, and caspase-1 and the activation of caspase-1.

### Antagonist of P2X7R could impede the creation of depressive-like behavior induced by CUS

**Fig. 4:**
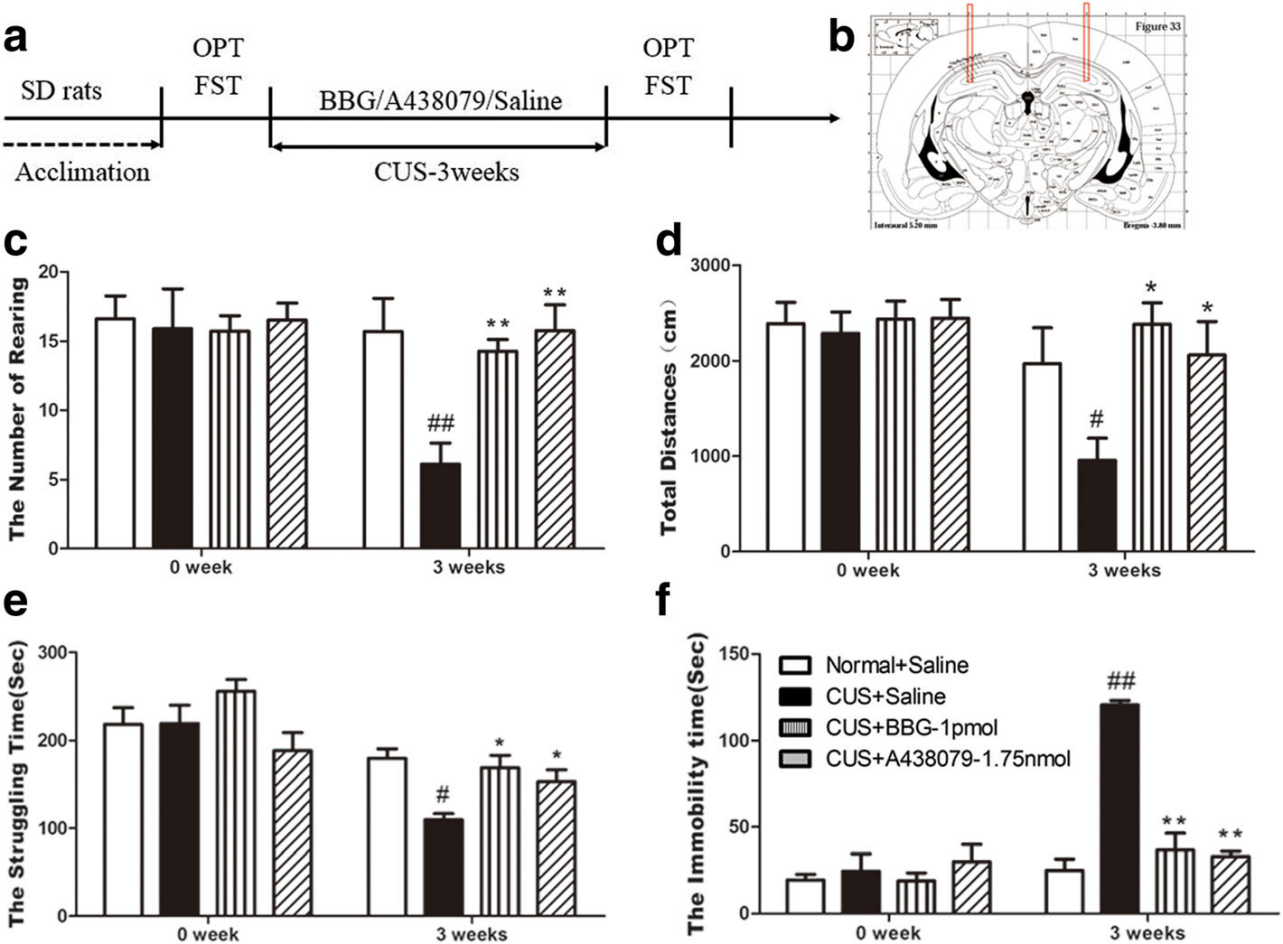
P2X7 receptor antagonism impeded the development of depressive-like behaviors induced by CUS. (**a**–**f**) Influence of A438079 or BBG on behavioral deficits induced by chronic unpredictable stress (CUS). **a** Experimental paradigm. Rats were randomly divided into four groups: normal + saline (*n* = 13), CUS + saline (*n* = 11), CUS + BBG (*n* = 11), and CUS + A438079 (*n* = 13). All rats in CUS groups were exposed to CUS with antagonist of P2X7 receptor (BBG, 1 pmol/rat; A438079, 1.75 nmol/rat) or vehicle administration in the hippocampus per day for 3 weeks. **b** A schematic representation of hippocampi sections. *Bars* indicate the placement of the guide cannulas (3.8 mm anteroposterior; ±3 mm mediolateral from bregma; 3.5 mm dorsoventral from the skull). Behavioral tests were then conducted, including **c** rearing numbers in open-field test (OFT) (*F*_*3,47*_ = 6.030, *p* < 0 .01), (**d**) total distance traveled in open-field test (OFT) (*F*_*3,47*_ = 3.518, *p* < 0.05), **e** struggling time in forced swimming test (FST) (*F*_*3,47*_ = 6.537, *p* < 0.05), **f** immobility time in forced swimming test (FST) (*F*_*3,47*_ = 60.660, *p* < 0.01). All data are expressed as the mean ± SEM. ^#^ *p* < 0.05, ^##^ *p* < 0.01, comparing to normal rats; *p < 0.05, **p < 0.01 compared to rats in CUS + saline group

To further clarify the role of P2X7 receptor in depressive-like behaviors, we investigated if P2X7R blockade could impede the formulation of depressive-like behaviors. For this aim, we treated the rats with CUS in combination with two types of antagonists of P2X7R, respectively (Fig. 4a, b). Compared to the normal group, the stressed rats exhibited a typical depressive-like behavior after 3 weeks of CUS, such as reduced rearing times and total distance traveled in OFT (Fig. 4c, d), and less struggle and extended immobility in FST (Fig. 4e, f). Meanwhile, the behavior deficits induced by CUS were blocked by hippocampal infuse with the antagonist of P2X7R, BBG, and A438079, respectively (Fig. 4c–f).

### CUS could not induce depressive-like behavior in P2X7-null mice

**Fig. 5:**
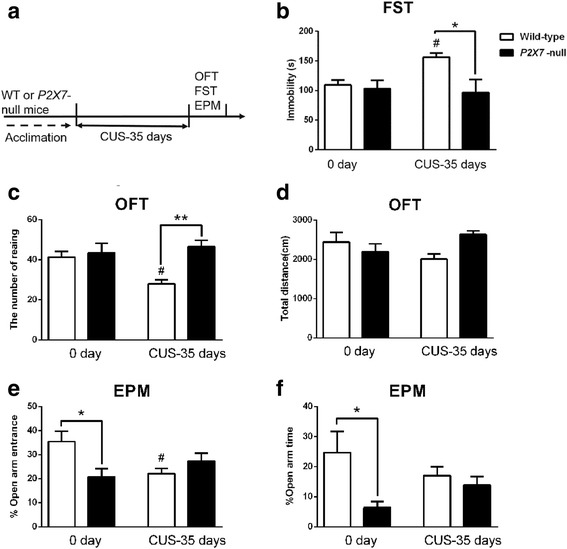
CUS induced depression-like behavior in wild-type C57BL6/J mice but not in mutant *P2X7-*null mice. **a** Experimental paradigm. Wild-type C57BL6/J (WT) and mutant *P2X7*-null mice were exposed to CUS for 35 days. Behavioral indicators were then assessed, including (**b**) immobility time in forced swimming test (FST) (interaction: *F*_*1, 37*_ = 4.501, *p* < 0.05; genotype: *F*_*1, 37*_ = 6.862, *p* < 0.05), **c** rearing numbers in open-field test (OFT) (interaction: *F*_*1, 37*_ = 6.204, *p* < 0.05; genotype: *F*_*1, 37*_ = 9.971, *p* < 0.01), (**d**) total distance in open-field test (OFT) (interaction: *F*_*1,37*_ = 4.918, *p* < 0.05), **e** Open-arm entrance percent in elevated plus maze test (EPM) (interaction: *F*_*1, 34*_ = 8.381, *p* < 0.01), and (**f**) open-arm time percent in elevated plus maze test (EPM) (genotype: *F*_*1, 34*_ = 5.194, *p* < 0.05). *n* = 8–12 per group; all data are expressed as the mean ± SEM. *p < 0.05 and **p < 0.01, comparing genotypes. ^#^ *p* < 0.05, comparing treatment

Based on the previous results, we further tested the wild-type male C57BL6/J and mutant male *P2X7-*null mice in CUS paradigms (Fig. 5a). The results show that CUS induced significant depressive-like behaviors in wild-type C57BL6/J mice, such as more immobility time in the FST and less rearing time in the OFT but not in *P2X7-*null mice (significant stressor treatment × genotype interactions) (Fig. 5b, c). Simultaneously, CUS stress did not affect both wild-type and *P2X7*-null mice locomotor activity in OFT (Fig. 5d). Interestingly, the *P2X7-*null groups reported greater symptoms of anxiety, regardless of whether being exposed to CUS [open-arm entrance percent (stressor treatment × genotype interactions); open-arm time percent (main effect of genotype)] (Fig. 5e, f). We also tested the female wild-type and female *P2X7*-null C57BL/6 mice in the same CUS paradigms. It was suggested that there was no significant difference between the sexes (Additional file 2: Figure S2).

### Chronic treatment with agonists of P2X7R-induced depressive-like behaviors

**Fig. 6:**
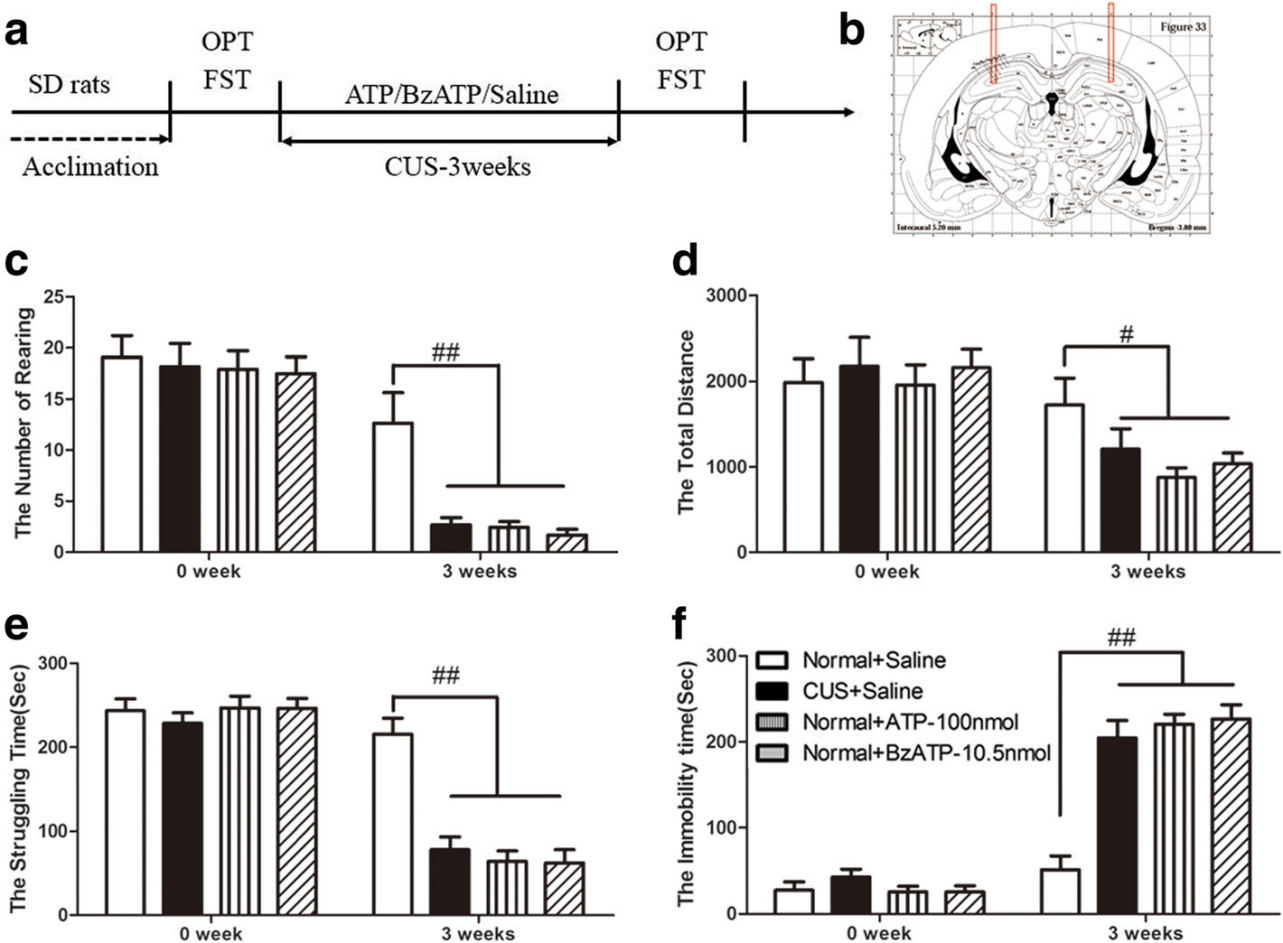
P2X7 receptor agonist administration induces depressive-like behaviors. **a**–**f** ATP or BzATP causes depressive-like behaviors which are the same as chronic unpredictable stress (CUS). **a** Experimental paradigm. Rats were randomly divided into four groups: normal + saline (*n* = 11), CUS + saline (*n* = 12), normal + ATP (*n* = 12), normal + BzATP (*n* = 12). All rats in CUS group were exposed to CUS with vehicle administration for 3 weeks; meanwhile, rats in ATP or BzATP group were administered with ATP (100 nmol/rat) or BzATP (10 nmol/rat), respectively, per day for 3 weeks. **b** A schematic representation of hippocampi sections. *Bars* indicate the placement of the guide cannulas (3.8 mm anteroposterior; ±3 mm mediolateral from bregma; 3.5 mm dorsoventral from the skull). Behavioral indicators were then assessed, including (**c**) rearing numbers in open-field test (OFT) (*F*_*3,46*_ = 11.502, *p* < 0 .01), (**d**) total distance traveled in open-field test (OFT) (*F*_*3,46*_ = 2.971, *p* < 0.05), (**e**) struggling time in forced swimming test (FST) (*F*_*3,46*_ = 20.868, *p* < 0.01), and (**f**) immobility time in forced swimming test (FST) (*F*_*3,46*_ = 24.357, *p* < 0.01) All data are expressed as the mean ± SEM. ^#^ *p* < 0.05, ^##^ *p* < 0.01 compared to normal rats

Since CUS can cause eATP to increase in the hippocampus and induce significant depressive-like behaviors, does ATP mediate the effect of stress on depressive-like behaviors via prolonged activation of P2X7R in rats? We further evaluated the effect of agonists of P2X7R (ATP and BzATP) in rats (Fig. 6a, b). As shown in Fig. 6, the rats showed decreased rearing times and reduced total distances in the OFT (Fig. 6c, d), and less struggle and more immobility in the FST (Fig. 6e, f) after being microinjected with ATP or BzATP for 3 weeks. This finding suggests that prolonged ATP or BzATP treatment causes the classic depressive-like behaviors.

## Discussion

The present study shows that stress accumulation causes an increase in extracellular ATP, the assembly of NLRP3 inflammasome, the cleavage of caspase-1, mature of IL-1β in the hippocampus as well as the depressive-like and anxiety-like behaviors in rodents. Hippocampal infuse of P2X7R agonist can cause significant behavioral deficits as exposure to CUS. In contrast, the behavioral deficits caused by CUS are impeded by pretreatment with a P2X7R antagonist or permanently knockout of P2X7R, suggesting that increased eATP activates P2X7R to mediate the development of behavioral deficits caused by CUS. Then, further studies are needed to clarify the source of extracellular ATP and to identify the receptors that mediate the release of ATP.

As we know, elevated extracellular ATP is necessary for P2X7R activation [56]. Only a very few studies [57–60] focuses on the change of eATP, endogenous ligand of P2X7R in any brain areas, although there are increasing studies that have demonstrated the important pathophysiological functions of P2X7R in CNS disorders, including neuropathic pain [61], trauma brain injury [62], and neurodegenerative illnesses [60, 63]. In recent years, there are only two papers that detected the levels of eATP in the hippocampus in animal model of depression [64, 65]. The results of these two papers are conflicted with each other. Inconsistent with the latter [64], our results present that eATP level was increased progressively in the hippocampus following chronic stress accumulation, although their results suggested that ATP is released during stress exposure [64]. And they also could not illuminate the sources of eATP. As they said, the neuron [66] and astrocyte [67] could be the source of eATP. Meanwhile, eATP, as a “warning molecule” or “danger signal”, is a trigger for microglial activation, and P2X7R could be the major mediator of this effect [68–71]. Even if the results confirmed the hypothesis, further studies should be done to clarify the precise mechanism which mediated stress-induced ATP release in the hippocampus.

**Fig. 7:**
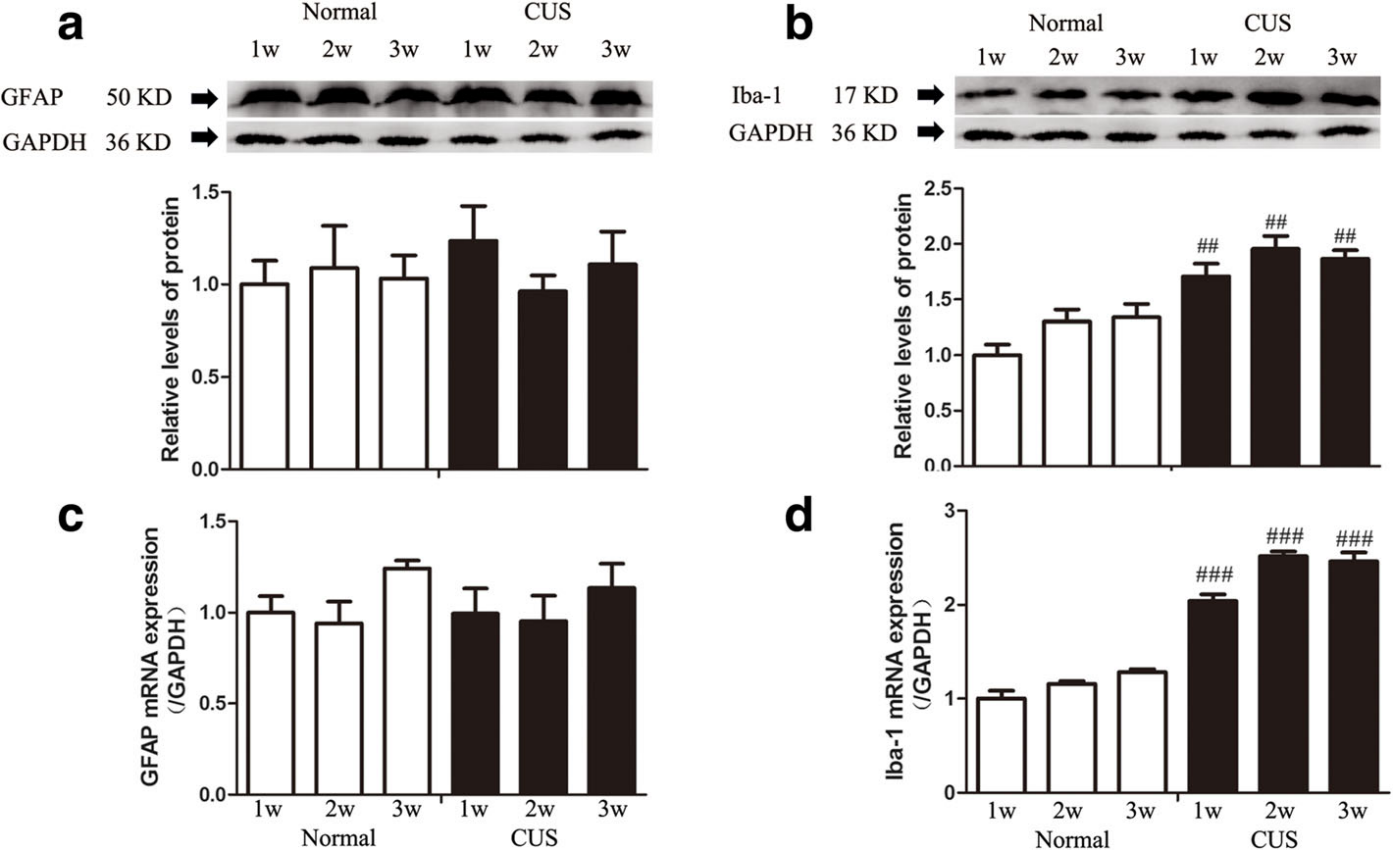
CUS could significantly increase the expression of Iba-1 in the hippocampus of rats in early stage. Western blots of GFAP (**a**) and Iba-1 (**b**) in the hippocampus. GFAP (**c**) and Iba-1 (**d**) mRNA level in different groups were expressed as a ratio to that of corresponding GAPDH. Results are expressed as the mean ± SEM (*n* = 4 per group). ^##^ *p* < 0.05, ^###^ *p* < 0.001 compared to normal rats at the same time point

There is strong evidence that neuroinflammation may play a key role in the development of affective disorders including depression and anxiety [7, 9]. In clinical, ischemia stroke [72], autoimmune multiple sclerosis (MS) [73], and some neurodegenerative diseases [74], which are characterized by significant neuroinflammation, have high rates of comorbidity with depression. A growing body of evidence has confirmed that the activation of microglia and overproduction of proinflammatory cytokines in some brain areas are associated with the development of these diseases [75–78]. Many studies of depression [79–82] have demonstrated peripheric inflammation activation, including elevated levels of IL-1β, IL-6 and interferon-γ (IFN-γ) in peripheral circulation in depressive patients or animal model of depression, but only several studies observed the increased concentration of IL-1β in some brain regions in animal model of depression[33, 83, 84]. In recent years, activated microglia was regarded as the source of local synthesized cytokines in brain. But, the research focus on microglia in depression presented paradoxical results [85–89]. Our results showed that following the stress accumulation, except interleukin-1beta level were increased, Iba-1, an activated marker of microglia were consistently increased, which hinting that microglia in the hippocampus exhibited an activated statement following chronic stress (Fig. 7). However, the activated type and precise role of microglia response to chronic stress merited further investigation.

As mentioned above, activated microglia could be the key source of neuroinflammatory cytokines, including IL-1β and the major mediator of many neuroinflammatory diseases [90, 91]. Consistently with some previous studies [92], our results exhibited that P2X7R is predominantly localized to microglia throughout the CNS. Thus, P2X7R may be responsible for stress-induced microglia activation. In addition, extracellular ATP-induced P2X7R activation could directly mediate K+ efflux which has been proposed to account for NLRP3 activation [93, 94]. Recently, several studies has also reported the change of NLRP3 inflammasome in rodents upon CUMS, LPS stimulus, or estrogen deficiency [95–97]. In their studies, they observed the overexpression of some components of NLRP3 inflammasome but did not detect the assembly of NLRP3 inflammasome. In accordance with their results, our results also showed that ASC, one of the components of NLRP3 inflammasome, are overexpressed. Except that we also observed that following the stress accumulation, the activation of NLRP3 inflammasome were increased, which were indicated by the oligomerization of NLRP3 with ASC and pro-caspase-1 and the cleavage f caspase-1. In accordance with our results, Ma et al. [98] indicated that NLRP3 primarily colocalized with microglia, providing further support for a tight relationship between P2X7R and NLRP3. Taken together, it implied that P2X7R may be responsible for chronic stress-induced neuroinflammation, possibly by NLRP3 inflammasome-dependent IL-1β mature and microglia activation.

In consistent with previous report [64], our results exhibited that chronic hippocampal infuse of two selective P2X7 antagonists, BBG and A438079, completely impeded the development of the depressive- and anxiety-like behaviors resulting from chronic stress. The similar effects have also been observed in P2X7R-null mice in this research as with the two earlier studies [27]. It has been reported that P2X7R antagonist rapidly blocks the induction of IL-1β by acute stress but does not produce a response in acute behavioral models [64]. In addition, previous studies have indicated that BBG inhibited the inflammatory response via the P2X7R/NLRP3 axis following intracerebral hemorrhage [99]. Combined with these previous findings, our results hinted that chronic stress causes eATP accumulation and sustained activation of P2X7R/NLRP3 axis. However, further research is merited to clarify whether the NLRP3 inflammasome is a key mediator of P2X7 blockage-induced anti-neuroinflammation and antidepressant-like effects.

## Conclusions

Chronic stress increases eATP level and then leads to neuroinflammation in the hippocampus, eventually leading to depression-like and anxiety-like behavior in rodents. P2X7R agonist also causes depression-like and anxiety-like behavior in rodents as chronic stress. In contrast, P2X7R blockage or null mutation of P2X7R is effective approach for blocking the deleterious effects or chronic stress on depressive and anxiety behaviors. Together, our data imply that P2X7/NLRP3 axis could be the potential therapeutic target for stress-induced affective disorders.

## Additional files

Additional file 1: Figure S1. Expression of P2X7 receptors in hippocampus. (A–I) Immunofluorescence staining of hippocampal sections from normal rats. P2X7R (green), Iba-1 (red), GFAP(red), DAPI(blue), × 40 objective; scale bar, 50 μm. (J) Percentage of P2X7 receptor-immunopositive cells. Three regions per hippocampus section and three sections per animal were counted by experimenters who were blind to the experiment design. (TIF 10328 kb)

Additional file 2: Figure S2. There was no significant sexual difference in the mice model of depression induced by chronic unpredictable stress. (A) Experimental paradigm. Wild-type C57BL6/J (WT) male and female mice were exposed to CUS for 35 days. Behavioral indicators were then assessed, including (B) immobility time in forced swimming test (FST) (interaction: F_1,34_ = 0.0003, *p* = 0.9857; stress: F_1,34_ = 26.51, *p* < 0.0001; sex: F_1,34_ = 0.4940, *p* = 0.4869), (C) the number of rearing in open-field test (OFT) (interaction: F_1,34_ = 0.01154, *p* = 0.9151; stress: F_1,34_ = 20.44, *p* < 0.0001; sex: F_1,34_ = 0.1414, *p* = 0.7092), (D) total distance in open-field test (OFT) (interaction: F_1,34_ = 0.03584, *p* = 0.8510; stress: F_1,34_ = 4.501, *p* = 0.0412; sex: F_1,34_ = 0.1341, *p* = 0.7165), (E) open-arm entrance percent in elevated plus maze test (EPM) (interaction: F_1,34_ = 0.1817, *p* = 0.6728; stress: F_1,34_ = 16.47, *p* = 0.0003; sex: F_1,34_ = 7.879, *p* = 0.0084), (F) open-arm time percent in elevated plus maze test (EPM) (interaction: F_1,34_ = 0.7491, *p* = 0.3932; stress: F_1,34_ = 5.100, *p* = 0.0309; sex: F_1,34_ = 0.2789, *p* = 0.6011) *n* = 8–12 per group, all data are expressed as the mean ± SEM. ^#^ *p* < 0.05, ^##^ *p* < 0.01, ^###^ *p* < 0.001, compared to male before CUS. *p < 0.05 and **p < 0.01, compared to female before CUS. (G) Experimental paradigm. Wild-type C57BL6/J (WT) and *P2X7*-null female mice were exposed to CUS for 35 days. Behavioral indicators were then assessed, including (H) immobility time in forced swimming test (FST) (interaction: F_1,23_ = 1.038, *p* = 0.3188; stress: F_1,23_ = 8.155, *p* = 0.0089; genotype: F_1,23_ = 1.610, *p* = 0.2171), (I) the number of rearing in open-field test (OFT) (interaction: F_1,23_ = 3.690, *p* = 0.0672; stress: F_1,23_ = 3.929, *p* = 0.0595; genotype: F_1,23_ = 1.221, *p* = 0.2805), (J) total distance in open-field test (OFT) (interaction: F_1,23_ = 4.348, *p* = 0.0483; stress: F_1,23_ = 0.2596, *p* = 0.6153; genotype: F_1,23_ = 2.684, *p* = 0.1150), (K) open-arm entrance percent in elevated plus maze test (EPM) (interaction: F_1,23_ = 5.294, *p* = 0.0308; stress: F_1,23_ = 2.595, *p* = 0.1208; genotype: F_1,23_ = 1.976, *p* = 0.1732), and (L) open-arm time percent in elevated plus maze test (EPM) (interaction: F_1,23_ = 5.914, *p* = 0.0232; stress: F_1,23_ = 3.100, *p* = 0.0916; genotype: F_1,23_ = 3.463, *p* = 0.0756). *n* = 5–8 per group, all data are expressed as the mean ± SEM. ^#^ *p* < 0.05, ^##^ *p* < 0.01, ^###^ *p* < 0.001, compared to wild-type before CUS. *p* < 0.05, comparing genotypes. (TIF 9760 kb)

## Abbreviations

ACSF: Artificial cerebrospinal fluid
ARL 67156 trisodium salt: 6-*N*, *N*-diethyl-β-γ-dibromomethylene-D-adenosine-5-triphosphate FPL 67156
CUS: Chronic unpredictable stress
IL-1β: Interleukin-1β
IP: Immunoprecipitation
P2X7R: P2X7 receptor
