# Supplementary figures and images for: Activation of P2X7 receptor and NLRP3 inflammasome assembly in hippocampal glial cells mediates chronic stress-induced depressive-like behaviors
Source: J Neuroinflammation. 2017 May 10;14:102. doi: 10.1186/s12974-017-0865-y (PMC5424302; doi:10.1186/s12974-017-0865-y)

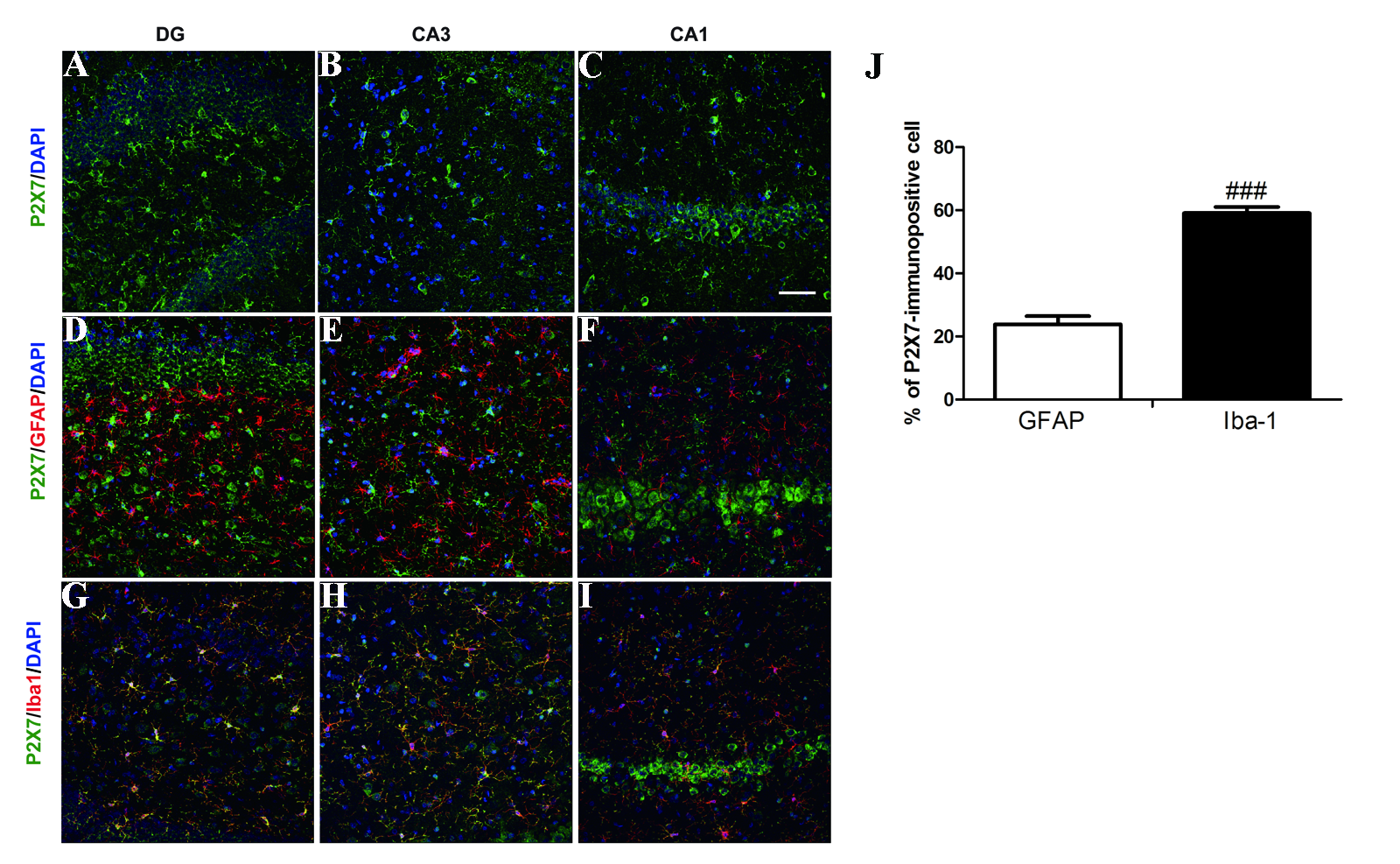

Supplement: Supplementary file 1 — Expression of P2X7 receptors in hippocampus. (A–I) Immunofluorescence staining of hippocampal sections from normal rats. P2X7R (green), Iba-1 (red), GFAP(red), DAPI(blue), × 40 objective; scale bar, 50 μm. (J) Percentage of P2X7 receptor-immunopositive cells. Three regions per hippocampus section and three sections per animal were counted by experimenters who were blind to the experiment design. (TIF 10328 kb) [file 12974_2017_865_MOESM1_ESM.tif]

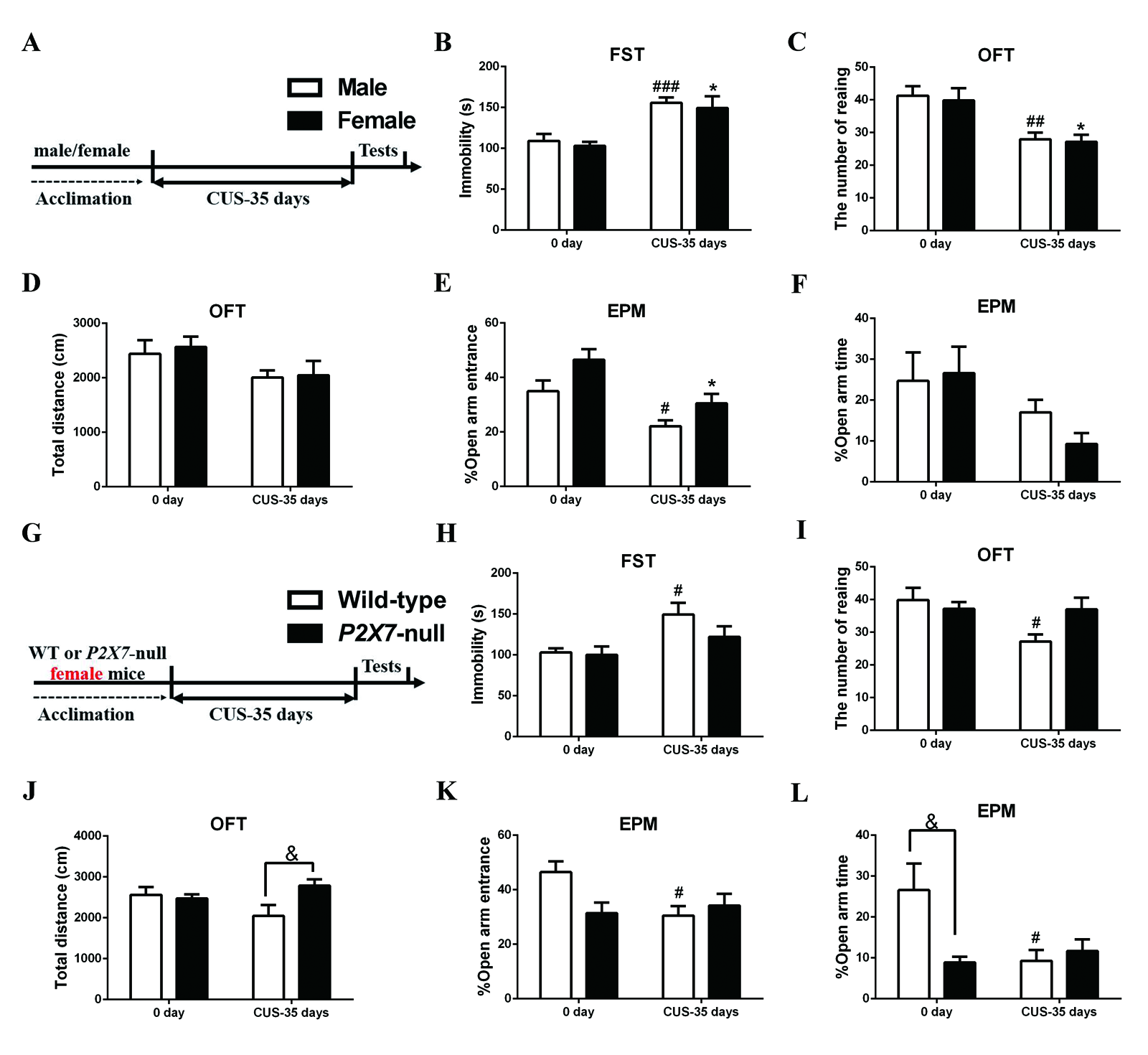

Supplement: Supplementary file 2 — There was no significant sexual difference in the mice model of depression induced by chronic unpredictable stress. (A) Experimental paradigm. Wild-type C57BL6/J (WT) male and female mice were exposed to CUS for 35 days. Behavioral indicators were then assessed, including (B) immobility time in forced swimming test (FST) (interaction: F1,34 = 0.0003, p = 0.9857; stress: F1,34 = 26.51, p < 0.0001; sex: F1,34 = 0.4940, p = 0.4869), (C) the number of rearing in open-field test (OFT) (interaction: F1,34 = 0.01154, p = 0.9151; stress: F1,34 = 20.44, p < 0.0001; sex: F1,34 = 0.1414, p = 0.7092), (D) total distance in open-field test (OFT) (interaction: F1,34 = 0.03584, p = 0.8510; stress: F1,34 = 4.501, p = 0.0412; sex: F1,34 = 0.1341, p = 0.7165), (E) open-arm entrance percent in elevated plus maze test (EPM) (interaction: F1,34 = 0.1817, p = 0.6728; stress: F1,34 = 16.47, p = 0.0003; sex: F1,34 = 7.879, p = 0.0084), (F) open-arm time percent in elevated plus maze test (EPM) (interaction: F1,34 = 0.7491, p = 0.3932; stress: F1,34 = 5.100, p = 0.0309; sex: F1,34 = 0.2789, p = 0.6011) n = 8–12 per group, all data are expressed as the mean ± SEM. # p < 0.05, ## p < 0.01, ### p < 0.001, compared to male before CUS. *p < 0.05 and **p < 0.01, compared to female before CUS. (G) Experimental paradigm. Wild-type C57BL6/J (WT) and P2X7-null female mice were exposed to CUS for 35 days. Behavioral indicators were then assessed, including (H) immobility time in forced swimming test (FST) (interaction: F1,23 = 1.038, p = 0.3188; stress: F1,23 = 8.155, p = 0.0089; genotype: F1,23 = 1.610, p = 0.2171), (I) the number of rearing in open-field test (OFT) (interaction: F1,23 = 3.690, p = 0.0672; stress: F1,23 = 3.929, p = 0.0595; genotype: F1,23 = 1.221, p = 0.2805), (J) total distance in open-field test (OFT) (interaction: F1,23 = 4.348, p = 0.0483; stress: F1,23 = 0.2596, p = 0.6153; genotype: F1,23 = 2.684, p = 0.1150), (K) open-arm entrance percent in elevated plus maze test (EPM) (int [file 12974_2017_865_MOESM2_ESM.tif]
